# The Intricate Link among Gut “Immunological Niche,” Microbiota, and Xenobiotics in Intestinal Pathology

**DOI:** 10.1155/2017/8390595

**Published:** 2017-10-08

**Authors:** Danilo Pagliari, Giovanni Gambassi, Ciriaco A. Piccirillo, Rossella Cianci

**Affiliations:** ^1^Department of Internal Medicine and Medical Sciences, “A. Gemelli Hospital”, Catholic University of the Sacred Heart, Rome, Italy; ^2^Department of Microbiology and Immunology, McGill University, Montréal, QC, Canada; ^3^Program in Infectious Disease and Immunity in Global Health, Research Institute of the McGill University Health Centre, Montréal, QC, Canada; ^4^Centre of Excellence in Translational Immunology (CETI), Research Institute of the McGill University Health Centre, Montréal, QC, Canada

## Abstract

Inflammatory bowel diseases (IBDs) are diseases characterized by various degrees of inflammation involving the gastrointestinal tract. Ulcerative colitis and Crohn's disease are characterized by a dysregulated immune response leading to structural gut alterations in genetically predisposed individuals. Diverticular disease is characterized by abnormal immune response to normal gut microbiota. IBDs are linked to a lack of physiological tolerance of the mucosal immune system to resident gut microbiota and pathogens. The disruption of immune tolerance involves inflammatory pathways characterized by an unbalance between the anti-inflammatory regulatory T cells and the proinflammatory Th1/Th17 cells. The interaction among T cell subpopulations and their related cytokines, mediators of inflammation, gut microbiota, and the intestinal mucosa constitute the gut “immunological niche.” Several evidences have shown that xenobiotics, such as rifaximin, can positively modulate the inflammatory pathways at the site of gut immunological niche, acting as anti-inflammatory agents. Xenobiotics may interfere with components of the immunological niche, leading to activation of anti-inflammatory pathways and inhibition of several mediators of inflammation. In summary, xenobiotics may reduce disease-related gut mucosal alterations and clinical symptoms. Studying the complex interplay between gut immunological niche and xenobiotics will certainly open new horizons in the knowledge and therapy of intestinal pathologies.

## 1. The Role of Mucosal Immunity in the Intestinal Mucosal Barrier

**Figure 1:**
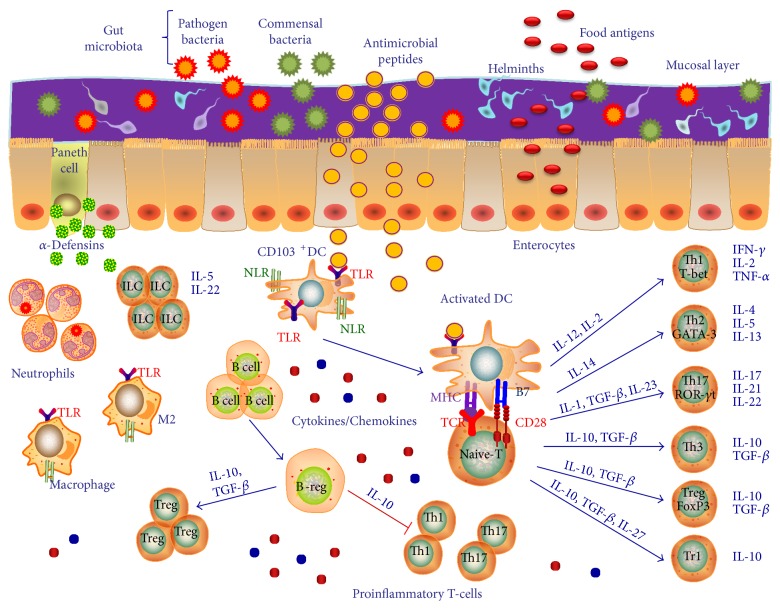
The complex interactions in the gut “*immunological niche*”. The human bowel is a sophisticated immune system that protects from pathogen's infections. The intestinal mucosal layer represents a mechanical barrier. The mucus over the gut epithelium contains antimicrobial peptides and it is the first defensive component. However, it is the intestinal *epithelium* with its secretory antibacterial peptides, innate and adaptive immune system cells, and their related pro- and anti-inflammatory cytokines and chemokines that regulate gut immunity. Intestinal mucosal immune cells are specifically organized to form a so-called gut-associated lymphoid tissue (GALT), where cells are activated by bacterial antigens. These structural and immunological defense mechanisms of the human gut have been referred to as the “*immunological niche*.” TLR: Toll-like receptors; Treg: regulatory T cells; NLR: NOD-like receptors; TCR: T cell receptor; IL: interleukin; GM-CSF: granulocyte-macrophage colony-stimulating factor; DC: dendritic cell; MHC: major histocompatibility complex; PD1: programmed death 1; PD-L1: programmed death-ligand 1.

Human bowel has a sophisticated immune system that protects from pathogen's infections, while maintaining a tolerance to food antigens and nonpathogen *bacteria* [1]. The mucus layer over the gut epithelium itself contains antimicrobial products and secretory IgA and it is the first defensive component. However, it is the intestinal *epithelium* with its secretory antibacterial peptides [2] and innate and adaptive immune system cells that regulates gut immunity (Figure 1). Intestinal mucosal immune cells are specifically organized to form a so-called gut-associated lymphoid tissue (GALT), where cells are activated by bacterial antigens. These structural and immunological defense mechanisms of the human gut have been referred to as the “mucosal firewall” [3].

It has been well established that CD4^+^ T cells can differentiate into several subtypes that may have both pro- and anti-inflammatory properties [4, 5]. Th1, Th2, and Th17 cells generate mucosal inflammation and tissue damage while regulatory T cells (Tregs), instead, have anti-inflammatory properties and limit mucosal inflammation and promote tissue repair. Thus, T cell subsets and their related cytokines contribute to the physiological maintenance and the pathological transformation of intestinal mucosa by constantly modulating the gut homeostasis and inflammation [6]. These T cell subpopulations present in the gut mucosa are associated with a specific cytokine cocktail [5]. Thus, several cytokines and their receptors with pro- and anti-inflammatory functions resulted involved in inflammatory diseases of the bowel [5, 7], such as IFN-*γ* and IFN-*γ*R1, TNF-*α*, IL-1R1, IL-2 and IL-2RA, IL-4, IL-5, IL-9, IL-12*β* and IL-12R, IL-13, IL-15, IL-17A, IL-18, IL-21, IL-22, IL-23 and IL-23R, TGF-*β* , IL-10, and IL-27 [8, 9]. The well-known proinflammatory T cell subpopulations are Th1 cells that are characterized by the specific production of IFN-*γ* and IL-12 and the counter-regulatory Th2 cells which produce humoral immunity-promoting cytokines like IL-4, IL-5, and IL-13. IL-12, a major product of activated DC, stimulates Th1 differentiation and production of effector cytokines, such as IFN-*γ* and TNF-*α*. As a classic activator of cell-mediated immunity, IFN-*γ* activates macrophages, natural killer (NK) cells, and CD8^+^ T cells. For Th2 cells, IL-4 acts as the major Th2 differentiation factor and promotes IL-4 and IL-13 expression. In particular, IL-13 is a cytokine with proinflammatory functions, as it participates in the disruption of gut epithelial barrier and in the promotion of mucosal fibrosis via TGF-*β*1 expression [10]. Furthermore, another proinflammatory T cell subset is constituted by Th9 cells characterized by the production of IL-9. IL-9 acts impairing gut mucosal healing, barrier function, and epithelial cell proliferation [11].

Th17 cells are key initiators of proinflammatory responses in gut mucosal surfaces. Th17 cells via their production of IL-17A and IL-17F are generally proinflammatory and play an important role in host defense against infection to extracellular pathogens, by recruiting neutrophils and macrophages to infected tissues. Their development depends on signals mediated by IL-6 (and downstream activation of STAT3) and TGF-*β*, IL-21, and IL-23 and by induction of the lineage-specifying transcription factor, retinoic acid-related orphan nuclear receptor (ROR*γ*T) [5]. IL-17A is involved in local chronic inflammation inducing proinflammatory cytokine expression leading to mucosal destruction and altering mucosal healing. Among Th17 cell cytokines, a key role is played by IL-23 that orchestrates the survival and maintenance of the Th17 phenotype and in turn the crosstalk between innate and adaptive immunity in the gut [12]. Interestingly, aberrant expression and activity of the IL-17/IL-23 axis is frequently involved in the pathogenesis of several inflammatory bowel pathologies [13, 14]. Consistently, studies report that polymorphisms in the *il23r* gene are associated with inflammatory bowel diseases (IBDs) [15, 16]. Another proinflammatory cytokine implicated in IBD pathogenesis is IL-21 which is secreted by T follicular helper (Tfh) cells, and it is implicated in the differentiation of germinal center B cells into high-affinity antibody-secreting plasma cells and memory B cells that ensure sustained immune protection and rapid recall responses against previously encountered foreign antigens [17]. IL-21 can also play important roles in T cell subset development. Tfh cells differentiate from naïve CD4^+^ T helper cell precursors after antigen activation in the presence of IL-6 and IL-21 and induction of B cell lymphoma 6 (BcL6) [4, 18]. It must be noted that while Th17 cells are prominent inducers of chronic inflammatory responses in disease states of the gut, this subset can protect the intestinal mucosa from microbiota and pathogens by its ability to resist infection and promote IgA secretion. Moreover, Th17 cells also produce IL-22 which has important functions in host defense at mucosal surfaces as well as in tissue repair [19]. While it is produced by innate lymphoid cells (ILCs) and Th cell subsets, including Th17 cells, IL-22 acts only on nonhematopoietic stromal cells like epithelial cells and keratinocytes. Although IL-22 is beneficial to the host in many infectious and inflammatory disorders, it can be pathologic due to its proinflammatory properties, which are further enhanced by other proinflammatory cytokines like IL-17 [20].

Several innate-like lymphocyte populations are involved in key homeostatic and pathogenic interactions with gut microbiota. Among these populations, a crucial role is played by ILCs, of which exist 3 subpopulations: ILC1, ILC2, and ILC3 [21]. These cells are part of the innate immune system, their actions are strictly related to the presence of commensal microbiota, and they interact between both the innate and adaptive arms of the immune system [22]. As it relates to the regulation of intestinal immune responses, ILC3 participates in the maintenance of mucosal barrier homeostasis by producing the anti-inflammatory cytokine IL-22, and they stimulate neutrophils and macrophage recruitment and proliferation in the gut, producing IL-17 and granulocyte-macrophage colony-stimulating factor (GM-CSF), respectively. In turn, macrophages may stimulate ILC function by producing IL-1*β* [23]. On the other hand, ILCs can stimulate T cells by favoring antigen presentation by intramucosal antigen-presenting cells (APCs), such as DCs [22].

The abovementioned cytokines and chemokines are principally related to several T cell subtypes of the adaptive immunity. Among these molecules, some of them have a bridge action in participating in both innate and adaptive immune response and include cytokines like IFN-*γ*, TNF-*α*, IL-1, IL-2, IL-12, and IL-15 [24]. Among these cytokines, a special role is played by IL-15 which plays key roles in the intestinal mucosal barrier [25]. IL-15 is a member of the IL-2 family of cytokines whose signaling pathway constitutes a bridge between innate and adaptive immune responses. On one hand, IL-15 regulates the differentiation and activation of proinflammatory Th1 and Th17 cells, and on the other hand, it blocks the activation of the immunosuppressive Tregs. In addition, IL-15 may mediate enterocyte apoptosis [26]. Consistent with these actions, several studies have reported an upregulation of IL-15 in inflamed tissues of patients with bowel pathologies, such as IBDs, and diverticular disease [27]. These data suggest that IL-15 may exert a direct pathogenetic role in these conditions. However, there is also evidence that IL-15 may potentiate the immune response against cancer. For these reasons, IL-15 is still considered both a friend and a foe of human physiology and pathology [28].

An essential role of the immune system is to eradicate pathogens while suppressing the potential for immune pathology. Triggering and maintaining immune tolerance within the intestine represent a unique challenge to the mucosal immune system. A variety of immune-regulatory cell subsets within the T cell, B cell, dendritic cells (DCs), and macrophage (M2 phenotype) compartments, each endowed with unique suppressive functions, are critical for ensuring sustained immune tolerance in the intestinal tissue microenvironment through active inhibition of innate and adaptive immune responses [20].

One of the predominant anti-inflammatory cell types are Tregs, of which there are many subtypes [29] including CD4^+^CD25^high^ Treg cells. These cells are characterized by the expression of the forkhead box P3 (FoxP3), the master-switch, lineage-specifying transcription factor that orchestrates the transcriptional landscape and drives the development and function of this Treg subset. FoxP3^+^ Tregs are essential mediators of immune tolerance by modulating innate and adaptive immune responses to self and nonself antigens [30]. Developmental, homeostatic, or functional deficits in these cells can provoke autoimmune disease and all the while augment responses to pathogens, tumors, or allergens [29]. Foxp3^+^ Tregs also have a positive role in limiting tissue inflammation, maintaining immune tolerance, and promoting mucosal healing in the gut [31]. In fact, studies have shown that their number is inversely correlated with the clinical course and severity of IBDs [6]. Moreover, the development of Tregs is strictly linked to the presence of commensal gut microbiota [32]. In fact, evidence in experimental mouse models showed that, in the absence of gut microbiota, the number of Tregs resulted significantly reduced and it was subsequently restored to normal proportions after gut recolonization with flora [33, 34].

DCs play an important role in activating immune responses but also in the induction of tolerance to microbial and dietary antigens [35]. DCs are environmental sentinels scanning for various innate and danger signals and poised in tissue to influence the immune activation or suppression decision. To this end, they are present in the mesenteric lymph nodes, in the gut *lamina propria*, and in Peyer's patches and participate in the control of intestinal inflammation [36]. Normally, DCs, particularly the tolerogenic CD103^+^ DC subset, are recruited in the gut during inflammatory conditions and in turn efficiently act stimulating the differentiation of Foxp3^+^ Tregs [37]. Overall, Foxp3^+^ Tregs, in concertation with other immunoregulatory cell types, including T regulatory 1 (Tr1) cells, T helper 3 (Th3) cells, regulatory B cells (Bregs), CD103^+^ DCs, and M2 cells, are instrumental in establishing a global context of immune tolerance to a spectrum of potentially pathogenic microorganisms in the gut flora [20].

## 2. The Gut Microbiota and Mucosal Immunity Crosstalk

Gut microbiota is the collection of all microbial populations that reside in the gastrointestinal tract. It can weigh up to a total of 1 kg and contains tens of trillions of microorganisms, a 100 times more genes than the host, and includes at least 1000 different bacterial species. It is increasingly recognized that gut microbiota plays a pivotal role in the gastrointestinal tract homeostasis [38]. A strong reciprocal interaction between gut microbiota and host immunity has been proven as the former has coevolved in a symbiotic relationship with mucosal immunity. These commensal *bacteria* are called “keystone species,” and overall, they can be considered a “superorganism” that is an integral part of the human gastrointestinal tract [39].

The human intestine has a large surface area that constitutes an entrance door for the antigens that we introduce with the food. In addition, the human gut is covered with many bacterial communities, some of which may be dangerous for the host. Hence, the principal role of the intestinal immune system is to protect the host from pathogens preventing infections. To this end, in mucosal immune cells, several surface receptors are endowed that mediate the interaction with microbial antigens. Among these pattern recognition receptors (PRRs), the most important are the Toll-like receptors (TLRs) and the NOD-like receptors (NLRs) [40].

In physiological homeostasis, there is a perfect balance between microbial load and the immune response generated against it [41]. The immune system correctly functions to ensure tolerance to food antigens and defense against microbial infections. A homeostatic role is played by TLR signaling. Commensal microbiota also participates to immune tolerance by promoting the differentiation of anti-inflammatory Tregs [42]. Conversely, in a disease state or during an infection, the normal gut homeostasis is lost with an excess of tissue inflammation. In this altered environment, TLRs, activated by pathogens, lose their homeostatic role and promote the activation and development of an inflammatory response, contributing to acute and chronic intestinal inflammatory states [43].

Host and microbial metabolisms are also key modulators of innate and adaptive immune responses in mucosal environments. While both occur simultaneously, the two are profoundly interdependent: while the host depends on the microbiome for a spectrum of digestive and metabolic enzymes, the microbiota, particularly in the gut, produces a wide array of metabolites from endogenous compounds produced by microbes and the host [44], but primarily from the anaerobic fermentation of dietary components in the colon [45]. The epithelial cell layer that constitutes the host microbe mucosal interface permits microbial-derived metabolic products to access and interact with host cells and, in turn, shape downstream inflammatory and immune responses. One salient example of such metabolite is short-chain fatty acids (SCFAs), like propionic acid and butyric acid, which are produced by colonic microbial fermentation of undigested or partially digested dietary fibres. They signal in host cells via G protein-coupled receptors (e.g., GPR41 and GPR43) on the surface of epithelial and immune cells, having a range of effects on host immune functions [46]. The effects of SCFAs include inhibition of histone deacetylase activity and altered gene expression in host cells and augmented epithelial barrier function which promotes gut homeostasis via several mechanisms: (1) enhanced mucus production by intestinal goblet cells, (2) inhibition of NF-*κ*B, (3) activation of inflammasomes and IL-18 production, (4) increased B cell secretion of secretory IgA, (5) diminished maturation of DCs, and (6) increased number and function of colonic Foxp3^+^ Tregs. SCFAs are not restricted to the gut and can also find their way to other organs, such as the lungs, where they directly or indirectly act on local APCs to modulate inflammatory responses that are associated with airway disease (allergic or infectious) [46–49].

## 3. Microbiota and Host Immune System Interactions in Inflammatory Bowel Diseases

IBDs, such as Crohn's disease (CD) and ulcerative colitis (UC), are chronic and multifactorial pathologies of the gastrointestinal tract. In these bowel diseases, there is an imbalance between proinflammatory and anti-inflammatory responses, but their full pathogenetic mechanisms are still incompletely understood [50]. It is known that in genetically predisposed individuals, an inappropriate immune response against luminal agents is activated with an abnormal production of cytokines and other mediators of inflammation [9]. The genetic factors involved in the pathogenesis of IBDs include genes encoding proteins of immunity involved in environmental sensing of microbial-derived products and signals.

In particular, nucleotide-binding oligomerization domain-containing protein 2 (NOD2) is an autophagy-related gene that activates an immune reaction against components of the bacteria cell wall, including peptidoglycans, in both Gram-positive and Gram-negative bacteria such as in the case of *Shigella* and *Listeria* [51]. NOD2 is expressed on the cell surface of various epithelial and innate immune cells, such as neutrophils, DCs, stromal cells, macrophages, and others [52]. NOD2 has several homeostatic functions like intracellular bacterial sensing, inducing the expression of several antibacterial peptides, such as *α*-defensin, and participating in the immune tolerance mechanisms by the suppression of the TLR axis [53]. Under physiological conditions, when the equilibrium between pro- and anti-inflammatory factors is maintained in the gut, NOD2 expression levels are very low [51]. On the other hand, in pathological situations, when proinflammatory factors are predominant over the anti-inflammatory ones, NOD2 level expressions are increased, particularly due to the overexpression of the proinflammatory cytokine TNF-*α* [51]. The activation of NOD2 by antimicrobial peptides determines the initiation of a signaling cascade which is responsible for the production of several proinflammatory cytokines and chemokines involving the activation of NF-*κ*B [52]. The breakdown of the physiological equilibrium between immunity and microbiota determines alterations in the NOD2 functions [54]. The bidirectional function of NOD2 is linked to the inflammatory levels present on the gut surface. Interestingly, NOD2 polymorphisms are associated with an increased risk for the development of CD, and aberrant NOD2 activity may alter the functions of both epithelial and innate immunity cells. Primarily, NOD2 in the epithelial compartment is able to selectively reduce the levels of alpha-defensins in Paneth cells in the small bowel [55, 56]. NOD2 activation is also linked to disturbance of hematopoietic cells and DC activity [57]. Moreover, NOD2 polymorphisms are related to a reduction of nuclear factor kappa-light-chain-enhancer of activated B cells (NF-*κ*B) in response to *bacterial* peptidoglycan [58]. Finally, other genetic factors involved in the pathogenesis of IBDs include the signal transducer and activator of transcription 3 (STAT3), and IL-23 receptor (IL-23R) [25].

Both humoral and cell mediated immunity are involved in the pathogenesis of IBDs [59]. IBDs are also characterized by an influence of environmental factors. In fact, IBDs pathogenesis is influenced by the TLR axis activated by both commensal and pathogenetic *bacteria*, leading to the promotion of inflammatory pathways responsible for tissue damage [25]. Likewise, IBDs are characterized by a profound modification of the gut microbiota. Typically, pathogens grow and proliferate to suppress the physiological *flora* and generate a dysbiotic state. The consequent intestinal barrier dysfunction determines the translocation of pathogens in the *lamina propria*. Human GALT is directly affected in at least two distinct ways. First, the outgrowth of opportunistic classes of *bacteria* drives increased inflammation [60]. In this scenario, TLRs mediate the activation of the proinflammatory transcription factor NF-*κ*B with the consequent production of proinflammatory cytokines and chemokines [61]. Secondly, the loss of benign fermenting *bacteria* that produce “keystone” metabolites results in reduced immunoregulation [3]. In particular, studies report that IBDs present specific gut microbiota alterations, characterized by a reduction in bacterial diversity and an increase in bacterial instability [13]. There is an increase of *Bacteroidetes* and *Proteobacteria* in both CD and UC [62, 63]. CD is further characterized by an increase in some pathogens of the *Enterobacteriaceae* family [64], such as *Salmonella*, *Shigella*, *Escherichia*, invasive *Fusobacteria*, and *Actinobacteria* [62]. Likewise, both CD and UC present a reduced number of *Firmicutes*, *Lachnospiraceae*, and *Ruminococcaceae* [65]. Finally, in CD, fewer *Bifidobacteria* have been reported [66].

Considering the immunopathogenesis of IBDs, it is well known that CD is related to a Th1 and Th17 immune profile, while UC is related to a Th2 response [67]. Then, both pathologies are characterized by the unbalance between proinflammatory T cell subsets and anti-inflammatory Tregs [59]. In fact, both in CD and UC, Tregs and their related anti-inflammatory cytokines resulted as decreased. Several literature data have reported a strong interaction among mucosal immunity, TLRs, and gut microbiota. These interactions may regulate gut physiology, immune tolerance to external dietary antigens, protection from external infections, regulation of gut microbial populations, and the commensal/pathogen ratio. A special focus has been placed on TLR2 and TLR4 signaling. In fact, their signals promote Treg proliferation and survival [68]. Then, other evidences supported the fact that such bacteria may directly influence the development of Th17 cell differentiation. In this way, *Bacteroides fragilis* promotes Treg differentiation and IL-10 and TGF-Beta production and inhibits Th17 cell differentiation [69].

## 4. Pharmacological Modulation of the Immunological Niche

These data suggest that the intestinal mucosa holds a complex immune-functional set that is central in the regulation of physiological homeostasis. For this reason, the gut mucosa may be considered an “immunological niche,” that is, a definite immune-functional region, that is, constituted by T cell subpopulations and their related anti- and proinflammatory cytokines, several mediators of inflammation, and gut microbiota [26, 70]. Perturbations and disruption of the immunological niche are critical steps in the pathogenetic pathways contributing to the development of inflammatory bowel pathologies. However, it is likely that we may also use the concept of immunological niche to explain the mechanisms of several other “inflammatory” diseases. An immediate therapeutic strategy would be to use agents that can modulate the immunological niche reducing inflammation and rebalancing gut immunity [71]. While there is no such definitely proven agent, there is a growing interest in the possible role of xenobiotics, especially rifaximin [72].

## 5. The Role of Xenobiotics in the Interactions with the Immunological Niche: The Case of Rifaximin

Xenobiotics are chemical molecules that are not normally produced by humans. They may interfere with host metabolism and produce effects of modification of the host physiology and pathology. Among xenobiotics, rifaximin seems to be of special interest [73]. Rifaximin is a semisynthetic agent based on rifamycin with a broad-action spectrum of antibiotic activity against both Gram^+^ and Gram^−^* bacteria*. Rifaximin has poor oral bioavaibility; thus, it acts locally in the gastrointestinal tract having only few systemic effects [74].

Differently from common antibiotics, rifaximin may modify gut microbiota toward a relative abundance of certain species of protective *bacteria*. Several data have confirmed that rifaximin is able to increase the proliferation and growth of the protective *Lactobacilli* while inhibiting that of several pathobionts, including *Clostridia* and *Firmicutes*, with negative effects [75, 76]. These changes in microbiota composition may contribute to the anti-inflammatory effects of rifaximin on the intestinal mucosa [77]. Indeed, *Lactobacilli* are able to downregulate mucosal inflammation, improving the function of intestinal barrier, and restoring the normal mucosal permeability [78].

**Figure 2:**
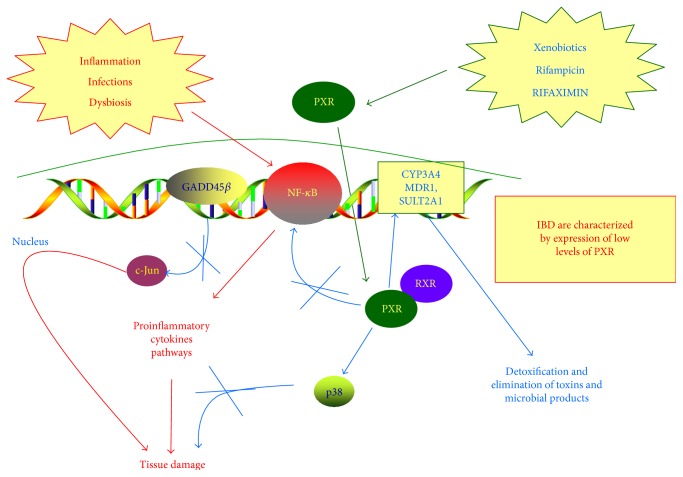
The role of rifaximin in modulating gut inflammation via the PXR/NF-*κ*B pathway. Rifaximin is an effective agonist of the nuclear receptor PXR. PXR, greatly expressed in liver and intestinal mucosa, acts as a driver of detoxification processes and contributes to intestinal cell survival during exposure to several xenobiotics. After being activated by its ligands, PXR translocates into the nucleus where it binds its receptor and then regulates DNA transcription. The anti-inflammatory effects of rifaximin may not only be linked to the reduction of ileal *bacteria* load but it may also have an indirect action on inflammation. In fact, rifaximin, being an effective agonist of PXR, may regulate the inflammatory process. In particular, rifaximin, activating PXR, can inhibit NF-*κ*B activity and the consequent transcription of several proinflammatory cytokines, such as TNF-alpha and IL-1*β*. The activation of PXR then upregulates p38 MAP kinase signal cascade via GADD45*β* upregulation. All these mechanisms are linked to the prevention of tissue damage and to favor gut mucosal healing.

The anti-inflammatory effects of rifaximin may not only be linked to the reduction of ileal *bacteria* load but it may also have an indirect action on inflammation. In fact, rifaximin is an effective agonist of the nuclear receptor PXR [79]. PXR, greatly expressed in liver and intestinal mucosa, acts as a driver of detoxification processes and contributes to intestinal cell survival during exposure to several xenobiotics. After being activated by its ligands, PXR translocates into the nucleus where it binds its receptor and then regulates DNA transcription (Figure 2). PXR can inhibit NF-*κ*B activity and the consequent transcription of several proinflammatory cytokines, such as TNF-alpha and IL-1beta [73, 80]. In 2010, Cheng et al. [81] demonstrated that rifaximin reduces the expression of the NF-*κ*B-related proinflammatory genes activating PXR function. In contrast, rifaximin is not able to modify the expression of these genes in PXR-*null* transgenic mice. For this reason, PXR is considered an anti-inflammatory molecular factor. In support of this, it has been proven that IBDs are characterized by the expression of low levels of PXR. Thus, rifaximin activation of PXR function may have positive anti-inflammatory properties. Finally, rifaximin may limit inflammation-mediated damage activating the p38 MAP kinase that is directly able to promote tissue repair [82].

## 6. The Role of Rifaximin in Inflammatory Bowel Diseases

It is well known that gut microbiota plays an important role in the development of IBDs [83]. Thus, the modulation of the gut microbiota has been put as focus of several clinical and research areas [84]. The manipulation of intestinal *bacteria* can be achieved by several modalities that involved xenobiotics, such as prebiotics, probiotics, and antibiotics, and fecal transplants [85, 86].

Prebiotics are fermentable substances participating in the modulation of gut flora. Prebiotics have several actions on the gut mucosa, such as the improvement of IL-10 DCs and TLR2 and TLR4 cells. Then, they exert positive modulation of gut microbiota populations improving the growth of beneficial resident *bacteria* as a consequence of the manipulation of the luminal substrate composition. Moreover, prebiotics improve the intestinal barrier and regulate the mucosal immune system [87].

On the other hand, probiotics are live microorganisms that, administered in therapeutic doses, confer a health advantage on the host. Probiotics present several positive actions on the gut mucosa. In fact, probiotics restore the microbial balance, protect the host against pathogens, and modify gut-associated lymphoid tissue and the mucosal immune system. A synergic action between prebiotics and probiotics seems to be associated with a reduction of the concentration of pathogenetic metabolites and dangerous microflora [85].

In particular, several antibiotics may modulate the course of IBDs by reducing pathological *bacteria*, such as *Escherichia coli*, *Bacteroides fragilis*, and other gram-negative *Enterobacteriaceae* present in the gut lumen, and by altering the composition of gut microbiota to favor beneficial *bacteria* [83]. Literature data demonstrated that antibiotics may be effectively used in the treatment of IBDs, due to their action in reducing bacterial overgrowth, resolving septic systemic complications and local infections, such as abscesses and fistulas [88]. Importantly, it has been shown that antibiotics may be used even to maintain IBD remission [89]. Among xenobiotics, rifaximin seems to influence remission in both CD and UC [90].

In 2005, Shafran and Johnson performed a clinical study on active CD patients. Rifaximin was administered at a dose of 200 mg twice daily for 16 weeks, and it induced a high clinical remission (59% cases) [91]. Then, in a further study, Shafran and Burgunder performed a retrospective analysis of CD patients receiving adjunctive therapy with rifaximin (mean dose 600 mg daily for 16 weeks) showing clinical remission in a high amount of cases (70%) [92]. In 2012, Prantera et al. performed a clinical trial comparing the twice-daily rifaximin administration of 400 mg, 800 mg, and 1200 mg versus placebo. This trial demonstrated that the administration of rifaximin 800 mg twice daily for 3 months was able to induce clinical remission of moderately active CD [93]. Surprisingly, the administration of rifaximin 1200 mg and 400 mg twice daily for 3 months had no significant higher induction of clinical CD remission versus placebo [93]. The treatment resulted safe and well tolerated by all patients. Furthermore, a similar trial on active, moderate CD reported significantly higher rates of remission after 12 weeks of treatment among patients receiving rifaximin 800 mg twice daily versus placebo. Thus, rifaximin may be used as an adjunct to standard therapy, although the authors did not address the surprisingly high clinical remission rates observed in both the rifaximin and placebo groups [94].

Differently from CD, data on the efficacy of rifaximin in UC are anecdotal [95]. In particular, in 1999, a clinical trial with rifaximin in unresponsive-to-steroids UC patients was conducted. This trial demonstrated that 400 mg rifaximin twice daily for 10 days significantly reduced clinical symptoms and mucosal healing in those patients [96]. In 2006, Guslandi et al. conducted a small clinical trial on 30 UC patients with steroid intolerance. Rifaximin 400 mg twice daily was added for 4 weeks to the mesalamine 2.4 g daily treatment, and clinical remission was obtain in a large amount of cases [97, 98]. In addition, it has been evaluated that the combined treatment with *Saccharomyces boulardii* 500 mg daily *plus* rifaximin 400 mg daily did obtain clinical remission in all mesalamine-resistant UC patients [99].

Recently, a clinical trial has been conducted with a fully humanized anti-IL-17A monoclonal antibody in CD patients [100]. However, this trial failed in this disease but it had satisfactory results in psoriasis [101]. In fact, literature data confirmed that the overexpression in the IL-17/IL-23 axis related to the increased level in Th17 cells constitutes a favorable prognostic factor in the pathogenesis of IBDs [6, 13].

Other innovative therapies for IBDs have been developed using humanized antibodies against the cytokines overexpressed in these diseases. To date, several anti-cytokine antibodies are approved for IBD therapy, including anti-TNFs (infliximab, etanercept, adalimumab, golimumab, certolizumab, etc.), anti-IL-17 (secukinumab, brodalumab), anti-IL-12/23 p40 (ustekinumab), and IL-23 p19 (tildrakizumab) [9].

## 7. The Role of Rifaximin in Diverticular Disease

A better understanding of the potential role of rifaximin is derived from a series of studies conducted by our group in patients with diverticular disease. In 2009, we demonstrated that in diverticular disease, there are several modifications in T cell subpopulations, both in peripheral blood and in colonic mucosa. In particular, these patients have an increased tissue recruitment of CD103^+^ lymphocytes [102]. These cells are characteristic of the intestinal homing, because they typically move from the peripheral blood to the gut mucosa [70].

It has been now well established that in patients with diverticular disease, rifaximin is able to ameliorate clinical symptoms reducing bacterial overgrowth and related mucosal chronic inflammation. Indeed, gut CD103^+^ lymphocytes are reduced after 2 months of rifaximin treatment. This is the first demonstration that rifaximin has the ability to not only modify gut microbiota and inhibit tissue inflammation but may also directly modulate T cell circulation and mucosal immunity. In 2014, we demonstrated that the number of TLR2 and TLR4 lymphocytes both in peripheral blood and in sigmoid mucosa is significantly altered in diverticular patients relative to controls [103]. An increased number of TLR2 and TLR4 cells in the peripheral blood of diverticular patients indicates an increase in activated circulating T cells. Moreover, after *placebo*, we demonstrated that the number of TLR2 and TLR4 lymphocytes increased. This evidence indicates that TLRs are indirect markers of bacterial overgrowth. These data reveal that rifaximin may act limiting bacterial overgrowth and then reducing the related TLR activation. TLRs mediate the activation of both innate and adaptive immune response and may also activate the proinflammatory transcriptional factor NF-*κ*B [43]. NF-*κ*B has a key role in the development of immune response against pathogenic *bacteria*. In fact, NF-*κ*B is associated with a transcription and secretion of a Th1 proinflammatory cytokine pattern [43]. In this way, these data demonstrate the effective role of rifaximin in modulating local and systemic TLR expression and T cell circulation and further confirm the well-established anti-inflammatory properties of this drug in addition to its antibacterial action [72]. Therefore, considering its multiple activities, rifaximin could be redefined as a “eubiotic” agent acting as a gut microenvironment modulator (Table 1).

## 8. Conclusions

Inflammatory bowel pathologies are a heterogeneous group of diseases characterized by various degrees of inflammation involving the gut mucosa. Several mediators of inflammation are involved in their pathogenesis, such as T cell subpopulations and their related pro- and anti-inflammatory cytokines, TLRs, and the pathogen/commensal ratio. A strong interaction among these factors has been well evaluated. In fact, on the one hand, gut microbiota may strictly modulate mucosal immunity, and on the other hand, mucosal immunity may influence the composition of gut microbiota. This complex interaction between gut microbiota and mucosal immunity is also mediated by other several factors that participate in the inflammatory pathways.

Inflammatory bowel pathologies are shared by the disruption of the physiological homeostasis present in the gut mucosa. First, the alteration in the pathogen/commensal ratio precipitates in the pathological condition of dysbiosis. During dysbiosis, the normal physiology of the gut mucosa is altered and there is bacterial translocation from the lumen to systemic circulation due to the leaky gut condition. In this pathological *scenario*, there is a dysregulation in the cytokine production, with the proliferation of proinflammatory T cell subsets and inhibition of anti-inflammatory ones. All these complex interactions among T cell subpopulations, gut microbiota, and the mediators of inflammation occur in the anatomical subset of the gut mucosa, which we have defined as “immunological niche.”

Several evidences have shown that xenobiotics may positively modulate the gut immunological niche. Most specifically, xenobiotics may interfere with components of the immunological niche, leading to an activation of anti-inflammatory pathways, and inhibition of several inflammatory mediators. As a result, xenobiotics may reduce disease-related gut mucosal alterations and clinical symptoms.

In summary, while further research is warranted, the complex interplay between gut immunological niche and xenobiotics has the potential to open new horizons in our knowledge of inflammatory bowel pathologies and their treatment.

## Figures and Tables

**Table 1:** The beneficial effects of the interaction between gut microbiota and immunological niche: the role of rifaximin as a “eubiotic” agent.

Rifaximin may act on both innate and adaptive immune cells and has a role on both mucosal and systemic immunity. Thus, it may have 3 levels of action	
(1) Gut microbiota	Positively selecting commensal gut microbial communities: (i) Increasing the proliferation and growth of the protective *Lactobacilli* (ii) Inhibiting the proliferation of several pathobionts, including *Clostridia* and *Firmicutes*

(2) Inflammation	Inhibiting the PXR-induced transcription of NF-*κ*B proinflammatory-related genes, such as TNF-alpha and IL-1beta

(3) Mucosal and systemic immunity	(i) Reducing TLR activation (ii) Interfering with T cell circulation and gut homing of CD103^+^ lymphocytes and inhibiting proinflammatory T cells, such as Th1 and Th17 cells
